# Simultaneous transient global amnesia and Takotsubo syndrome after death of a relative: a case report

**DOI:** 10.1186/s13256-018-1928-0

**Published:** 2019-01-25

**Authors:** Josef Finsterer, Claudia Stollberger

**Affiliations:** 1 Krankenanstalt Rudolfstiftung, Postfach 20, 1180 Vienna, Austria; 2 2nd Medical Department with Cardiology and Intensive Care Medicine, Krankenanstalt Rudolfstiftung, Vienna, Austria

**Keywords:** Broken heart syndrome, Stress cardiomyopathy, Memory, Catecholamines, Heart failure, Stunned myocardium

## Abstract

**Introduction:**

Simultaneous occurrence of transient global amnesia and Takotsubo syndrome has been only rarely reported. Here we report another patient with a transient global amnesia and concomitant Takotsubo syndrome.

**Case presentation:**

Our patient is a 64-year-old white man with a previous history of myocarditis from borreliosis who developed sudden-onset confusional state with perseverations and repetition of the same questions during a funeral for his brother-in-law. Upon neurological work-up and after spontaneous resolution of most of the neurological deficits, transient global amnesia was diagnosed. Blood tests revealed moderate renal insufficiency, elevated troponin-T, and elevated N-terminal prohormone of brain natriuretic peptide. Electrocardiography showed left anterior hemiblock and negative T-waves in V2–V6. Upon transthoracic echocardiography the apical type of a Takotsubo syndrome was suspected. Since coronary angiography was normal and electrocardiography and echocardiographic abnormalities resolved under candesartan, bisoprolol, acetyl-salicylic acid, and atorvastatin within a few days after onset, Takotsubo syndrome was diagnosed.

**Conclusions:**

Since Takotsubo syndrome may be associated with transient global amnesia a causal relation may exist. A possible trigger for both conditions could be severe emotional stress from the loss of a close relative. A possible common pathomechanism could be overstimulation of adrenergic receptors in the myocardium, the cerebrum, or the coronary or cerebral arteries. Whether pre-existing myocardial compromise promotes the development of Takotsubo syndrome requires further investigations.

## Introduction

Simultaneous occurrence of transient global amnesia (TGA) and Takotsubo syndrome (TTS) has been only rarely reported (Table 1) [1–9]. Whether TGA triggers TTS in such a case or vice versa, or whether there is a common trigger for both conditions is so far unsolved. Here we report a tenth patient, the first male, with the simultaneous occurrence of a TGA and TTS.

**Table 1:** Patients with simultaneous transient global amnesia and Takotsubo syndrome so far reported

Age	Sex	TRI	DTGA (hours)	TTTS	THER	RECG (days)	RECHO (d)	OC	Reference
72	f	Emotional stress	nm	Classic	nm	nm	nm	CRC	[19]
77	f	Assault	16–24	Classic	None	nm	5	CRC	[20]
69	f	Worries	24	nm	PM	nm	nm	CRC	[5]
62	f	Swimming	18	Classic	None	nm	nm	CRC	[4]
66	f	Sister’s death	~ 48	Classic	None	nm	6	CRC	[21]
57	f	Son’s death	24	Classic	nm	nm	nm	CRC	[3]
63	f	nm	~ 24	Classic	BB, ACEI	nm	nm	CRC	[22]
60	f	Death anniversary	16	Classic	None	nm	4	CRC	[1]
57	f	Worries	< 24	Classic	None	nm	days	CRC	[23]
65	m	Death of relative	~ 24	Classic	HFT	nm	nm	CRC	Current case

*ACEI* angiotensin-converting inhibitor, *BB* beta-blocker, *CRC* complete recovery, *DTGA* duration of transient global amnesia, *f* female, *HFT* heart failure therapy, *m* male, *nm* not mentioned, *OC* outcome, *PM* temporary pacemaker, *RECG* recovery of electrocardiogram in days after onset, *RECHO* recovery of echocardiography in days after onset, *THER* therapy, *TRI* trigger, *TTTS* type of Takotsubo syndrome

## Case presentation

**Fig. 1:**
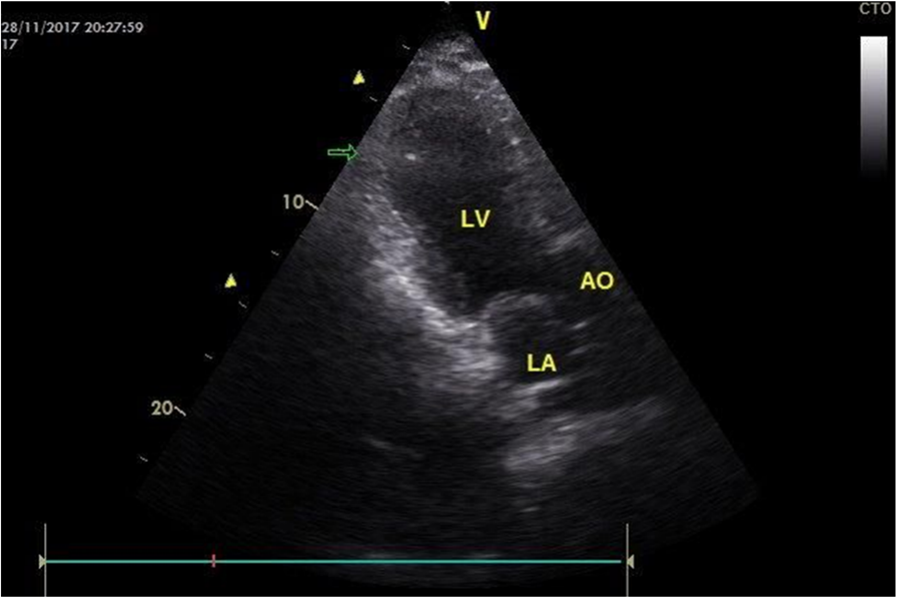
Transthoracic echocardiography. Endsystolic apical five-chamber view on hospital day 1 showing dyskinesia of the left ventricular posterior, posterolateral and apical parts. *AO* aorta, *LA* left atrium, *LV* left ventricle

Our patient is a 64-year-old white man, height 176 cm, weight 90 kg, who developed a sudden-onset confusional state with perseverations and repetition of the same questions during a funeral for his brother-in-law to whom he had a close emotional relation. He had a previous history of arterial hypertension, myocarditis due to borreliosis with systolic dysfunction that was diagnosed 13 years prior to the current admission, and an allergy to penicillin. He was regularly taking candesartan and bisoprolol. A clinical neurologic examination on admission revealed disorientation in all qualities, retrograde amnesia, and reduced tendon reflexes but was otherwise normal. Blood pressure on admission was 140/77 mmHg. An electrocardiogram (ECG) showed left anterior hemiblock and negative T-waves in V2–V6. Blood tests revealed moderate renal insufficiency, high-sensitive troponin-T of 243 ng/L (normal, < 14 ng/L), and an N-terminal prohormone of brain natriuretic peptide (NT-proBNP) of 588 ng/L (normal, < 241 ng/L). MRI of his cerebrum was normal. Transthoracic echocardiography revealed dyskinesia of the left ventricular posterior, posterolateral, and apical parts of the left ventricular myocardium and apical ballooning (Fig. 1). Clinical cardiologic examination was normal. On hospital day (hd) 2 his troponin-T fell to 77 ng/L. An electroencephalogram (EEG) was normal. Coronary angiography on hd4 was normal but ventriculography still showed mild apical ballooning. The neurological manifestations of the stress syndrome resolved except for mild memory disturbances for some words within a few hours after onset. Echocardiography and ECG normalized under medication with candesartan, bisoprolol, acetyl-salicylic acid, and atorvastatin within a few days after onset. Cardiologic and neurologic follow-up investigations 6 weeks after onset of the clinical manifestations were normal.

## Discussion

TGA is characterized by a sudden episode of memory loss that cannot be attributed to a more common neurological condition, such as epilepsy or stroke [6]. During a TGA, recall of recent events vanishes: the patient cannot remember where he is or how he got there. In addition, the patient may not remember anything about what is happening in the here and now [6]. Consequently, the patient keeps repeating the same questions because he does not remember the answers he has just been given. He may also draw a blank when asked to remember things that happened a day, a month, or even a year ago [6]. The cause of TGA is unknown but some studies indicate that ischemia in the hippocampus and the thalamus [7], migraine-related mechanisms, venous flow abnormalities (abnormal venous drainage from the temporal lobes), epileptic phenomena, or psychological disturbances [8] could be causative. Risk factors for developing a TGA may be a migraine history, cardiovascular risk factors, and emotional stress. Since TGA may be associated with TTS in single cases (Table 1) [1–5], it has been recently proposed that TGA could also be due to a catecholamine storm and could represent the cerebral form of a TTS [2]. However, none of these speculations has been proven to consistently explain the common occurrence of TGA and TTS [9]. Occasionally, TGA may be associated with troponine elevation [10]. Cortisol secretion can be increased during a TGA [11]. Cerebral MRI patterns may indicate that seizures trigger the development of a TGA [12]. Recently, cytotoxic edema of the hippocampus has been related to the pathogenesis of a TGA [13]. Fluorodeoxyglucose-positron emission tomography (FDG-PET) studies revealed that a TGA is associated with decreased metabolism in the posterior medial network [14]. Compensatorily, metabolism within the anterior temporal network is increased [14].

TTS is a peculiar, acute-onset type of regional systolic dysfunction together with regional dyskinesia, akinesia, or hypokinesia of the left ventricular wall [15] often resembling an acute coronary syndrome clinically, electrocardiographically, and laboratory-chemically, affecting predominantly postmenopausal women after a stressful trigger [16]. In general, four types of TTS can be delineated: the classical apical-midventricular type, the midventricular type, the basal type, and the global type. Although coronary angiography needs to be normal per definition, occasionally TTS is associated with coronary heart disease. The cause and pathogenesis of TTS remain controversial but is has been speculated that adrenergic overstimulation of the myocardium may result in a decrease of contractility. Hormonal influences and innate susceptibility may play modifying roles in the pathogenesis of TTS [17]. Involvement of the autonomic nervous system may contribute to the development of TTS [18]. Patients with TTS either receive treatment or do not. Treatment includes heart failure therapy and eventually antiarrhythmic medication or a pacemaker/implantable cardioverter defibrillator (ICD) implantation. The ECG resolves within 10 weeks after onset and the echocardiographic abnormalities within 6 weeks after onset. The outcome is fair in the majority of cases but some patients die from intractable heart failure, particularly those who experience the global type or complications.

Concerning the current case, the trigger of TTS seems to be emotional stress during the funeral of a close relative. Whether TTS occurred before the TGA, after the TGA, or simultaneously with the TGA remains speculative, because no cardiologic examination was carried out immediately at onset of the TGA. Possibly, systolic dysfunction from the TTS resulted in cerebral ischemia or a venous drainage problem and thus caused the memory problem. However, it cannot be excluded that overstimulation of cerebral adrenergic receptors in neurons or vascular smooth muscle cells led to the memory deficits. Concerning this speculation, further investigations are warranted. Concerning the trigger in the current case, the close relation between our patient and the deceased person suggests that strong emotions of mourning and grief may have been the initiating event. Concerning the triggers in the nine previously reported cases of TTS and TGA they were always emotional stress. Concerning the type of TTS, all previously described patients presented with the classical type (Table 1), Among these nine patients treatment for TTS was applied in two (Table 1). The outcome was favorable in all cases so far reported.

### Established facts

Simultaneous occurrence of TGA and TTS has been only rarely reported.

### Novel insights

Since TTS may be associated with a TGA a causal relation may exist.

A possible common pathomechanism could be overstimulation of adrenergic receptors in the myocardium, the cerebrum, or the coronary or cerebral arteries.

A trigger for both conditions could be severe emotional stress from loss of a close relative.

## Conclusions

This case shows that TTS may be accompanied by a TGA suggesting that a causative relation could exist between these two conditions. A possible trigger for both conditions could be severe emotional stress from the loss of a close relative. A possible common pathomechanism could be overstimulation of adrenergic receptors in cardiomyocytes, neurons, or vascular smooth muscle cells. Pre-existent cardiomyopathy may facilitate the development of TTS. Also males can be affected by TTS and TGA.
